# Interobserver agreement of estimating the extent of intestinal metaplasia in patients with chronic atrophic gastritis

**DOI:** 10.1007/s00428-021-03245-9

**Published:** 2021-12-13

**Authors:** Julia M. Lerch, Rish K. Pai, Ian Brown, Anthony J. Gill, Dhanpat Jain, Bence Kővári, Ryoji Kushima, Kieran Sheahan, Tomas Slavik, Amitabh Srivastava, Gregory Y. Lauwers, Cord Langner

**Affiliations:** 1 Diagnostic and Research Institute of Pathology, Diagnostic and Research Center for Molecular BioMedicine, Medical University of Graz, Neue Stiftingtalstraße 6, 8010 Graz, Austria; 2 Department of Laboratory Medicine and Pathology, Mayo Clinic Arizona, Scottsdale, AZ USA; 3 Envoi Specialist Pathologists, Brisbane, Queensland Australia; 4 Faculty of Medicine, University of Queensland, Brisbane, Queensland Australia; 5 Cancer Diagnosis and Pathology Group, Kolling Institute of Medical Research, Royal North Shore Hospital, St Leonards, New South Wales Australia; 6 New South Wales Health Pathology, Department of Anatomical Pathology, Royal North Shore Hospital, St Leonards, New South Wales Australia; 7 Sydney Medical School, University of Sydney, Sydney, New South Wales Australia; 8 Department of Pathology, Yale University School of Medicine, New Haven, CT USA; 9 Department of Pathology, University of Szeged, Szeged, Hungary; 10 H. Lee Moffitt Cancer Center & Research Institute, Tampa, FL USA; 11 Department of Pathology, Shiga University of Medical Science, Shiga, Japan; 12 Department of Pathology & Centre for Colorectal Disease, St. Vincent’s University Hospital, Dublin, Ireland; 13 University College Dublin, Dublin, Ireland; 14 Ampath Pathology Laboratories, Pretoria, South Africa; 15 Department of Anatomical Pathology, Faculty of Health Sciences, University of Pretoria, Pretoria, South Africa; 16 Department of Pathology, Brigham and Women’s Hospital, Boston, MA USA

**Keywords:** Chronic atrophic gastritis, Intestinal metaplasia, Interobserver agreement, Gastric precancerous lesion

## Abstract

The extent of gastric intestinal metaplasia (GIM) can be used to determine the risk of gastric cancer. Eleven international gastrointestinal expert pathologists estimated the extent of GIM on haematoxylin and eosin (H&E)- and Alcian blue-Periodic acid Schiff (AB-PAS)-stained slides of 46 antrum biopsies in 5% increments. Interobserver agreement was tested with the intraclass correlation coefficient (ICC). Correlation between standard deviation and extent of GIM was evaluated with the Spearman correlation. The interobserver agreement was very good (ICC = 0.983, 95% confidence interval (CI) 0.975–0.990). The use of AB-PAS did not increase the agreement (ICC = 0.975, 95% CI 0.961–0.985). Cases with a higher amount of metaplastic epithelium demonstrated a higher standard deviation (rs = 0.644; *p* < 0.01), suggesting lower diagnostic accuracy in cases with extensive GIM. In conclusion, estimating the extent of GIM on H&E-stained slides in patients with chronic atrophic gastritis can be achieved satisfactorily with high interobserver agreement, at least among international expert gastrointestinal pathologists.

## Introduction

Gastric atrophy (GA) and gastric intestinal metaplasia (GIM) have been identified as precancerous lesions suitable for risk stratification for gastric cancer. Different staging systems which require, at minimum, biopsies of both antrum and corpus, have been proposed to define the extent of GA and GIM, such as the Operative Link for Gastritis Assessment (OLGA) [1] or the Operative Link on Gastric Intestinal Metaplasia (OLGIM) [2].

For individuals with extensive GIM, defined as GIM involving both antrum and corpus, an approximately twofold increased risk of neoplastic progression was reported compared to individuals with limited GIM [3]. While individuals with OLGIM III–IV are at high risk of early gastric neoplasia, individuals with OLGIM II were denominated as intermediate risk patients [4].

Notably, the yield of GIM may be higher when multiple biopsies are sampled, indicating that patients with a higher total number of biopsies may have a higher probability to be classified as patients with extensive GIM, that is, as “patients at risk” [5]. Therefore, scoring systems that are independent of the number of biopsies have been applied, referring either to the relative number of biopsies involved by GIM [6] or to the percentage of mucosa involved by GIM [7–9]

Our study, which includes a group of international expert gastrointestinal pathologists, aimed to evaluate the interobserver agreement in estimating the overall percentage of mucosa involved by GIM and to identify parameters with potential impact on the assessment.

## Material and methods

### Cases

The study included antral biopsies from 46 patients (20 females and 26 males with a mean age of 65.8 years; median 69, range 27–87) with chronic atrophic gastritis, diagnosed at the Institute of Pathology, Medical University of Graz, Austria. All biopsies had been obtained based on Sydney criteria, that is, targeting the lesser and greater curvature, excluding the normal gastroduodenal transitional mucosa. It may be of note that corpus and/or fundus biopsies, which had been submitted in separate vials, lacked GIM in all cases and were therefore not part of the evaluation.

Since Austria is a country with a low prevalence of *Helicobacter pylori*, resulting in a low incidence of GIM in general (with a low proportion of mucosal surface involved), we selected the study sample in order to enrich for cases with a high amount of GIM. All samples were stained with haematoxylin and eosin (H&E) and with Alcian Blue-Periodic acid Schiff (AB-PAS) and were scanned thereafter (Pannoramic 1000 Whole-Slide Scanner, 3D Histech Ltd., Budapest, Hungary).

### Pathologists

Eleven international expert gastrointestinal pathologists participated in the study. Access to scanned slides was provided by an electronically transferred web link. The assessment was performed independently (blinded to endoscopic data) on dynamic images (3D Histech Ltd. Case Viewer, Budapest, Hungary). Specifically, the pathologists were asked to estimate the overall percentage of mucosa involved by GIM in 5% increments, that is, across all biopsies included within a given sample.

### Statistical analysis

Numerical variables are presented as mean, median and range. The interobserver agreement was assessed by applying the intraclass correlation coefficient (ICC), which is used to measure the degree of agreement for continuous variables for different observers when assessing the same cases [10]. The calculation is based upon a two-way mixed model and absolute agreement. For interpretation, the scheme introduced by Altman (1991) was used: an ICC value ≤ 0.20 suggests poor agreement, 0.21–0.40 fair agreement, 0.41–0.60 moderate agreement, 0.61–0.80 good agreement, and > 0.80 very good agreement, respectively [11].

Correlation between the standard deviation and the extent of GIM, defined by the mean score of GIM of the eleven observers, and the number of biopsy pieces per slide was evaluated by applying the Spearman correlation. Finally, a regression analysis was performed to establish a model that allows the prediction of the standard deviation from the extent of GIM. To account for non-consistent scattering in our dataset, we performed adjustment with heteroscedasticity-consistent standard error estimators [12].

All statistical operations were performed using IBM SPSS Statistics Version 26, provided by the Medical University of Graz. *P*-values were two-sided, and values < 0.05 were considered statistically significant.

## Results

### Interobserver agreement

The mucosa of the 46 cases was involved by GIM in different quantities, with mean values of 29% (range 6.8–82.7%) for H&E-stained and 25% (range 6.4–81.7%) for AB-PAS-stained slides. Mean values for individual observers ranged from 23.3 to 33% for H&E-stained and 15.3 to 33.8% for AB-PAS-stained slides. The interobserver agreement was very good, with an ICC value of 0.983 (95% confidence interval (CI) 0.975–0.990) for H&E-stained slides and 0.975 (95% CI 0.961–––0.985) for AB-PAS-stained slides, respectively. Thus, the use of AB-PAS did not increase agreement. Table 1 shows the interobserver correlation matrix of the eleven pathologists for H&E-stained slides.

**Table 1:** Interobserver correlation matrix for H&E-stained slides

	#1	#2	#3	#4	#5	#6	#7	#8	#9	#10	#11
#1	1.000	0.966	0.920	0.958	0.882	0.923	0.965	0.874	0.830	0.947	0.742
#2	0.966	1.000	0.927	0.953	0.871	0.944	0.949	0.901	0.817	0.952	0.717
#3	0.920	0.927	1.000	0.916	0.846	0.882	0.915	0.880	0.790	0.934	0.679
#4	0.958	0.953	0.916	1.000	0.869	0.902	0.934	0.894	0.778	0.924	0.749
#5	0.882	0.871	0.846	0.869	1.000	0.851	0.860	0.893	0.669	0.851	0.692
#6	0.923	0.944	0.882	0.902	0.851	1.000	0.930	0.866	0.802	0.911	0.691
#7	0.965	0.949	0.915	0.934	0.860	0.930	1.000	0.876	0.804	0.961	0.723
#8	0.874	0.901	0.880	0.894	0.893	0.866	0.876	1.000	0.725	0.901	0.677
#9	0.830	0.817	0.790	0.778	0.669	0.802	0.804	0.725	1.000	0.814	0.722
#10	0.947	0.952	0.934	0.924	0.851	0.911	0.961	0.901	0.814	1.000	0.674
#11	0.742	0.717	0.679	0.749	0.692	0.691	0.723	0.677	0.722	0.674	1.000

### Correlation between the standard deviation and potential impact factors

The number of biopsy pieces per slide ranged from 1 to 6 (mean 2.7, median 2). No correlation between the standard deviation and the number of biopsy pieces was observed on H&E-stained slides (*p* = 0.059). The six cases with the lowest standard deviation had a mean biopsy number of 3.0 (median 2.5, range 2–6) whereas the six cases with the highest standard deviation had a mean biopsy number of 2.3 (median 2.5, range 1–4).

**Fig. 1:**
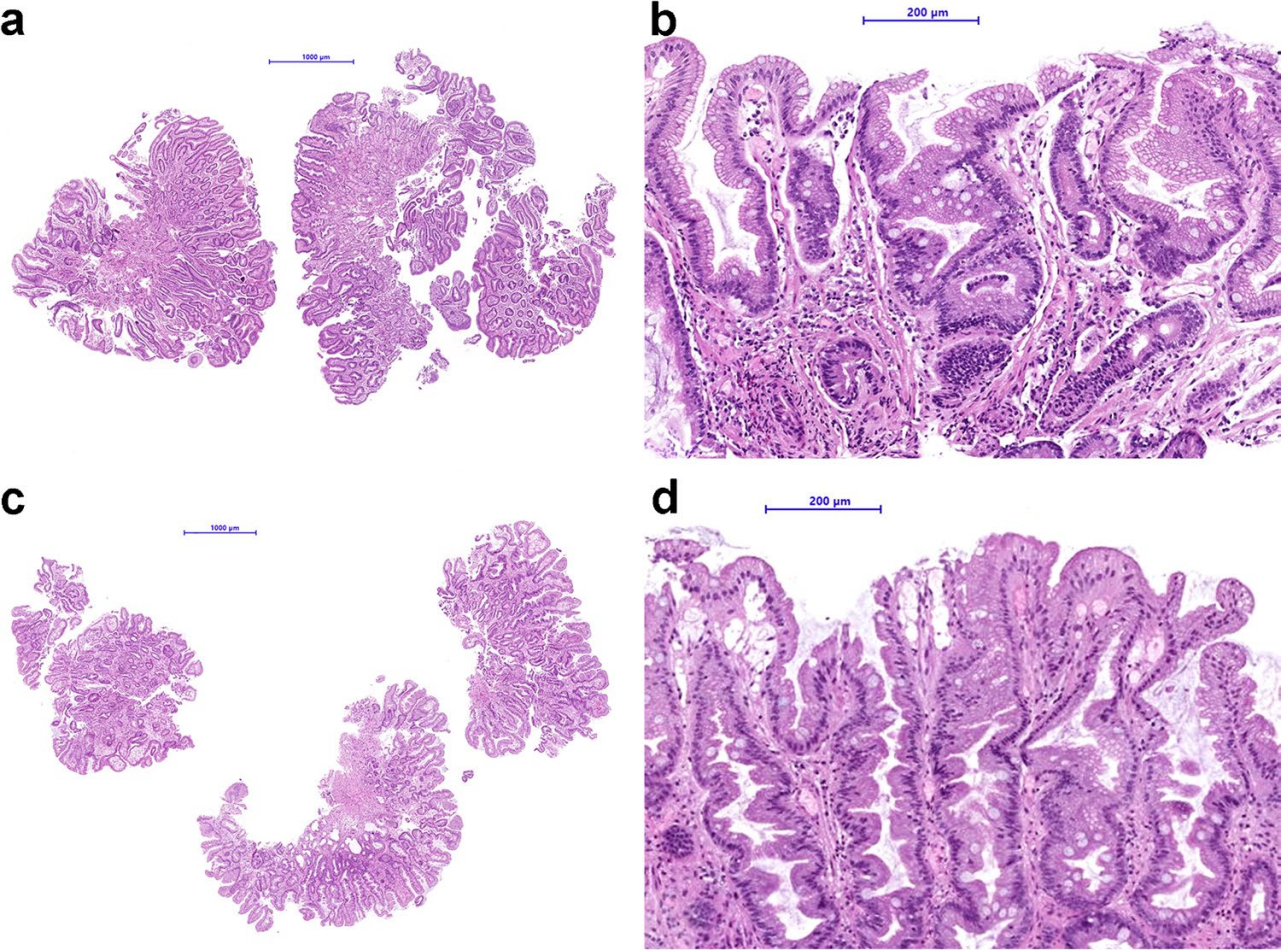
Histology of a case with high interobserver agreement (mean extent of gastric intestinal metaplasia of 10.5%, standard deviation of 4.2%) at low (a) and high (b) magnification. Histology of a case with low interobserver agreement (mean extent of gastric intestinal metaplasia of 67.7%, standard deviation of 25.5%) at low (c) and high (d) magnification

**Fig. 2:**
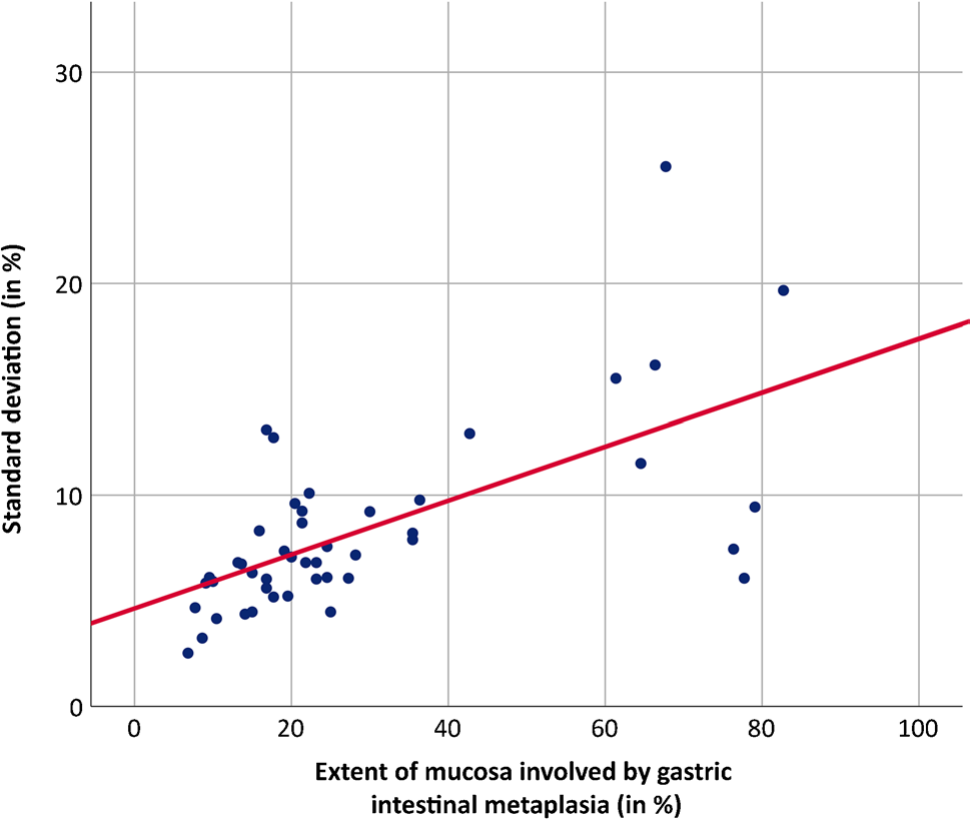
Correlation between the extent of gastric intestinal metaplasia (GIM assessed on H&E-stained slides, in %) and the standard deviation (SD, in %): The SD increases with the amount of metaplastic epithelium identified on the mucosal surface (SD = 4.568 + 0.127 × extent GIM)

Cases with a higher amount of metaplastic epithelium had a higher standard deviation. The significant positive association between both parameters was verified by applying the Spearman correlation, rs = 0.644 (*p* < 0.01). A simple linear regression was calculated to predict the standard deviation based upon the extent of GIM. According to the regression equation with *R*^2^ of 0.403, the standard deviation increased by 0.127 (*t* = 2.862, *p* < 0.01) for each percent of the extent of GIM (F(1,44) = 29.749, *p* < 0.01; Figs. 1 and 2).

## Discussion

The extent of GIM in patients with chronic gastritis has prognostic relevance and identifies patients at higher risk of gastric cancer who could benefit from endoscopic surveillance [3] Current guidelines by the European Society of Gastrointestinal Endoscopy (ESGE) [13], the British Society of Gastroenterology (BSG) [14], and the American Gastroenterological Association (AGA) [15] classify the extent of GIM as “extensive,” when GIM affects both antrum and corpus, requiring, at minimum, biopsies from both regions of the mucosa. For patients with extensive GIM endoscopic surveillance is recommended, while patients with GIM limited to the antrum do not need follow-up [13–15]

The number of biopsies may affect the histological diagnosis, that is, the yield of GIM may increase when multiple biopsies are obtained [5] In addition, biopsy specimens may be involved in varying quantities, with some showing only tiny foci of GIM, whereas others may show total replacement of the original mucosa. Consequently, some authors suggested alternative scoring methods, such as the (relative) number of biopsies involved by GIM [6] or the percentage of mucosa involved by GIM [7–9]

Our study proves for the first time that pathologists can estimate the extent of GIM on a semi-quantitative scale with very good agreement. The use of AB-PAS did not improve the agreement reached on H&E-stained slides. Therefore, this stain cannot be recommended to estimate the extent of GIM for routine practice. The standard deviation did not correlate with the number of biopsy pieces per slide, indicating that semi-quantitative assessment in 5% incremental steps can be applied irrespectively of the number of sampled biopsies.

It is of note, however, that the standard deviation increases with the amount of GIM, suggesting lower diagnostic accuracy in cases with extensive GIM. High amounts of GIM, i.e., involvement of several biopsy pieces within one sample and/or multiple foci within a single biopsy piece, may impede the estimation and may thereby have a negative impact on the quality of assessment.

Our study has strengths and limitations. Strengths include the systematic approach involving a large international group of gastrointestinal expert pathologists who analysed a large set of biopsies showing different quantities of GIM. Some may regard the lack of an independent “gold standard,” e.g., provided by morphometric image analysis, as a limitation of the study. We regarded this, however, as outside the scope of our project, in particular since the routine assessment of GIM is done by usual light microscopy and not by morphometry or comparable tools. Another limitation might be the use of virtual microscopy, which bears specific technical challenges: pathologists may find it harder to move around all biopsy specimens with the same ease they do on a microscope. However, the findings in our study are still relevant in view of the expected increase in the use of virtual diagnostics in the future.

In conclusion, estimating the percental extent of GIM on H&E-stained slides in patients with chronic atrophic gastritis can be achieved satisfactorily with high interobserver agreement, at least among international expert gastrointestinal pathologists. Our brief report provides the basis for future research in the field, e.g., by expanding the evaluation to general pathologists in a nation-wide setting, and for potential implementation of percental GIM assessment in the respective guidelines on gastric precancerous lesions.

## Data Availability

The datasets generated during and/or analyzed during the current study are available from the corresponding author on reasonable request.
